# Aggressive bodyguards are not always the best: Preferential interaction with more aggressive ant species reduces reproductive success of plant bearing extrafloral nectaries

**DOI:** 10.1371/journal.pone.0199764

**Published:** 2018-06-27

**Authors:** Bruno Gabriel Melati, Laura Carolina Leal

**Affiliations:** 1 Programa de Pós-graduação em Ecologia e Evolução, Departamento de Ciências Biológicas, Universidade Estadual de Feira de Santana, Novo Horizonte, Bahia, Brazil; 2 Departamento de Ecologia e Biologia Evolutiva, Universidade Federal de São Paulo, Diadema, São Paulo, Brazil; Universidade de Sao Paulo Faculdade de Filosofia Ciencias e Letras de Ribeirao Preto, BRAZIL

## Abstract

Variation in partner species and frequency of interaction between species pairs are potential drivers of the net outcome of generalized mutualisms. In ant-plant mutualisms, the quality of defence provided by ants is related to ant aggressiveness. Hence, we hypothesize that the performance of plants bearing extrafloral nectaries will be higher when they interact more frequently with more aggressive ant species. We estimated ant aggressiveness in the field by observing their behaviour towards soil baits. Afterwards, we observed the frequency with which individuals from these ant species visited plants through an entire reproductive cycle. We measured the production and persistence of plants reproductive structures through this period and the total seed production. Increasing in the interaction frequency with highly aggressive ants reduced the number of floral buds and seeds produced. Increased visitation frequency by less aggressive ants increased the number of floral buds and seeds per branch. The inverse relationship between ant aggressiveness and seed production may be influenced by the costs imposed by different mutualistic partners. Thus, frequent interaction with highly aggressive ants may lead to a higher accumulation of costs through time, resulting in a negative net outcome for the plants. Our results bring new evidence highlighting the importance to incorporate temporal aspects in the study of mutualistic interactions. We suggests that the quality of mutualistic partners must be understood as a function of its per-interaction benefit and their cumulative costs to their partner over time, what puts in check our current classification regarding partner quality in mutualistic systems.

## Introduction

Mutualisms are positive interspecific interactions in which the net outcome is determined by the interplay between the costs and benefits of the interaction for the individuals of both interacting species [1]. In generalized/facultative mutualisms, a species interacts with several partner species, instead of having a specialized interaction with a single partner species [2]. In this case, the costs and benefits of the generalized mutualism for the individuals of a given species are determined by the interaction between each individual and individuals from all potential partner species available in the habitat [3]. Consequently, it is possible that, in local scale, the net outcome of generalized mutualism for each individual is determined by the net benefit of interacting with each partner species available in the habitat and by the frequency of interaction between each different species pairs over time [4]. These two components have been evaluated as potential factors affecting mutualism outcome in empirical studies, however, its effects have been seldom evaluated jointly (e.g.[4–6], but see [7–9]). This approach contributes to a simplified view about the forces driving the net outcome of generalized mutualisms, once it neglects the cumulative effect of interactions with different partner species over time on individual’s fitness.

Regarding generalized mutualisms, the intrinsic quality of different species of mutualistic partners available in a habitat can be strongly variable [3,10–12]. This variation in the quality of different mutualistic species has been commonly related to differences in species morphological, behavioural or physiological traits that determine the quality of the services or rewards provided to their partners (e.g.,[13,14]). Thus, species that possess traits that allow them to grant higher net benefit per interaction for its partners are classified as high quality mutualistic partners [3,15, 16]. However, species within a community vary not only in the traits mediating its quality as a mutualistic partner, but also in their population sizes and spatial distribution (e.g.,[17,18]). These last variables directly influence the frequency with which partner species may interact with one another in the habitat (e.g.,[18]). The higher the frequency with which two species interact, the higher will be the influence of each species on the net outcome of the generalized mutualism for the other [4]. Hence, it is expected that individuals interacting more frequently with high quality partners will benefit more from the mutualism than those interacting more frequently with low quality partners [19–21].

One of the most studied examples of generalized mutualism is the interaction between plants bearing extrafloral nectaries (EFNs) and ants [22]. Extrafloral nectaries are part of the indirect anti-herbivory defence mechanisms used by plants, since EFNs attract predacious arthropods, mostly ants, which patrol the plants and protect them against herbivores (see [23]). Despite these ant-plant interactions being generally accepted as mutualisms, the efficiency of plant defence against herbivores and the plant fitness increment as a consequence of such defence are apparently highly variable in both space and time (e.g.,[4,23,24]).

Because EFNs are easily accessible structures, they attract a wide array of ant species that markedly differ in their behaviour, especially in their aggressiveness [25]. Within ant communities, aggressiveness is a trait directly related to species competitive ability, mainly because it determines ant species behaviour towards other co-occurring ants [26]. More aggressive ant species are also those that exhibit high competitive ability, mostly due to its capacity to forage in groups and defend resources, leading to a numerical and behavioural monopolization of resources in the environment [27]. These dominant ant species strongly influence the behaviour of the subordinate ones that exhibit lower competitive ability, and usually forage and defend resources individually [26]. These subordinate ants commonly exhibit low recruitment of other individuals from the colony and rarely monopolize resources [27,28]. Due to their behavioural characteristics, dominant species have been frequently pointed out as high-quality partners in protective mutualisms [29–31]. Therefore, if the net benefit of this ant-plant mutualism can be determined by the interaction between the quality of the defence provided by different ant species and the frequency of interaction between different species pairs, EFN-bearing plants that are more frequently visited by dominant ants should benefit more as they will be more efficiently defended against herbivores over time than those interacting mostly with subordinate ant species [25,31].

Here, we investigate how variation in the frequency of EFN visitation by ants with varying degrees of aggressiveness influences the reproductive success of EFN bearing plants. We hypothesize that the higher the frequency of interaction with more aggressive ant species, the higher will be the reproductive success of plants bearing EFNs. To evaluate our hypothesis, we used as study model the herbaceous plant *Turnera subulata* (Turneraceae) and the assemblage of ants visiting its EFNs in a semi-arid ecosystem in Brazil.

## Material and methods

### Study site and species

We conducted our study in a patch of Caatinga vegetation inside the campus of the State University of Feira de Santana, Northeastern Brazil, between December 2014 and March 2015. Caatinga vegetation is characterized as a mosaic of xerophytic, deciduous, thorn shrubs and seasonally dry forests occupying more than 880 000 km^2^ of Northeastern Brazil [32]. Our study area has a semi-arid climate with annual mean temperature of 25.2°C and mean annual precipitation of 848 mm. In our study site, there are a few EFN-bearing species, including the herbaceous *Turnera subulata* (Turneraceae) which we used as model species in this study due to its high local abundance. This species is a ruderal plant, commonly found in disturbed habitats such as roadsides and abandoned areas [33]. It exhibits a pair of EFNs on the underside of its leaves, in the junction between the petiole and the leaf blade. The extrafloral nectar is easily accessible, so that visiting ants do not need any specific behavioural or morphological adaptations to access this resource.

### Ant aggressiveness measures

In the literature, ant species are broadly classified into three hierarchical dominance categories: top dominants, second order dominants and subordinates [26–28]. This classification is based on ant species ability to defend and monopolize resources into a worldwide comparative view among different ant species. However, ant ability in monopolizing resources does not depend only on their own intrinsic capabilities but is also on the behavior of other co-occurring ant species [26]. In communities in which top dominant species are absent, for example, ant species classified as sub-dominant can play the same functional role of top dominant species (e.g. [34–37]) and so act as a high-quality bodyguard to plants bearing EFN in this habitat. Therefore, in order to identify ant species that are more aggressive in our study site, locally acting as dominant species and providing a better defensive service to EFN-plants, we measured local ant species aggressiveness towards other ant species when sharing feeding resources on the ground. As our model plants are prostate herbaceous plants occurring in an open habitat, ant species foraging on the ground are the most common species attending its EFNs (Leal, L.C., personal observation). Therefore, ant aggressiveness towards food resources on the ground should be a good proxy of ant aggressiveness when foraging on the plants.

We selected three large areas (~2,200 m^2^ each) in which *T*. *subulata* occurs naturally within our study site and in which our focal plant would be selected. In each of these areas, we placed four baits of canned sardines with honey over cardboard squares of 10 x 10 cm. We placed each set of four baits in a 30 m transect, so that each bait was 10 m away from the closest bait. To improve the reliability of our aggressiveness measure, we offered these baits for five consecutive days in each area twice per day during the periods of highest ant visitation on EFNs in the study site (06:00–08:00h e 16:00–18:00 h) (Melati, B., personal observation), so that the most common EFN-attending ants should be active during these periods.

We inspected the baits continuously through the two hours during which they were active and registered only the interactions occurring over the cardboard squares. At each inspection, we performed three minutes observation during which we counted the number of individuals of each ant species exploiting the baits. When individuals from two or more species were present at the same time at the cardboard squares, we extended the period of observation to five minutes to observe the frequency of aggressive behaviours between individuals from different species. We considered that ants where performing aggressive behaviour whenever individuals from one species shoved, bit, chased or sprayed formic acid against individuals from another species. We quantified the aggressiveness degree of each ant species as the number of times we observed individuals from that species behaving aggressively against other ants divided by the total number of times in which we observed the species exploiting the baits during the five days of observations. This metric allowed us to place the ant species occurring in the study site along a gradient of aggressiveness that can be used as a proxy of local ant dominance and, consequently, as proxy of ant species quality as plant bodyguard. Since individuals can exhibit more than one aggressive behaviour per observation, aggressiveness values can be higher than one. Thus, the higher the number of aggressive behaviours exhibited by each ant species per observation, the higher the aggressiveness value of the ant species and the greater must be the ant species aggressiveness towards potential plant herbivores.

### Influence of ant visitation over plant reproductive success

To evaluate if variation in the frequency of interaction between EFN-bearing plants and ant species with varying degrees of aggressiveness affects the interaction outcome for the plants, we inspected 191 individuals of *T*. *subulata* in the same three large areas mentioned above. From now on, we will refer to these plants as focal plants. Since *T*. *subulata* has clonal reproduction and grows in dense aggregations, we considered each aggregate of branches sprouting from the same point in the soil as a single individual. To determine the frequency with which focal plants were attended by ants, determine main ant visitor identity, and measure the plant reproductive success, we observed the focal plants for 22 consecutive days. During the first five days of observation, we performed the observations both in the morning and in the afternoon (07:00–09:00h and 16:00–18:00h). In each observation, we registered the presence and identity of the ant species visiting the EFNs of each focal plant. At the end of these five initial days of observation, we noticed that we registered the same set of ant species in each plant in both periods of observation, even though the proportion of plants attended by ants was higher in the morning than in the afternoon (morning: 0.64 and afternoon: 0.35). Thus, in the following 17 days we only followed ant visitation on the focal plants during the morning. The period of 22 days of observations allowed us to follow a complete reproductive cycle of all focal plants, from flower budding to seed setting. At the end of our observation period, therefore, we obtained the frequency with which ants in general visited each focal plant and the proportion of those visits performed by each ant species. We are aware that the reproductive success measures obtained through this reproductive cycle result mostly from previous interactions between the focal plants and the local ant assemblage. However, as ants and plants are both sessile organisms, their probability of interaction is mainly determined by their spatial distribution [38]. As we have no reason to believe that plant and ant species distribution on our study site have changed in the recent years, we argue that the visitation frequency we measured is a good estimate of the previous pattern of ant visitation on the focal plants in our study area.

To measure the reproductive success of focal *T*. *subulata* individuals, we randomly chose the most apical flowering branch per plant. In the individuals with a single flowering branch, we choose this single branch. We marked and counted the floral buds on each chosen flowering branch. During the daily observations, we counted the number of buds that developed into flowers, the number of flowers that developed into fruits, and the number of fruits that ripened. We considered as ripe fruits the ones that were not aborted during fruit development and that have produced seeds. Finally, we collected all ripe fruits and counted the number of seeds.

### Data analysis

At the end of our observations, we got all information about ant visitation and reproductive performance from 110 focal plants. The additional 81 plants marked at the beginning of our observations were excluded from the analysis because the plants were heavily damaged by the fall of trees or branches from nearby trees.

Since mutualistic partners with higher interaction frequency over time are those that most influence the reproductive success of their partners [4], we considered the ant species most commonly observed on each focal plant as the plant's main ant visitor. In all cases, we considered as the main ant visitor the ant species observed at least 60% of the time in which each focal plant had been attended by ants. Therefore, any increasing in the proportion of visits of focal plants by ants in general was directly related to an increase in the frequency of interaction between the focal plant and its main ant visitor. Using the aggressiveness measures taken in the field, we grouped these morphospecies in three categories according to their aggressiveness in soil baits: ants with low, intermediate and high aggressiveness. We performed this grouping due to the low number of plants attended by some of these ant species (see the results), which could have limited our ability to correlate each plant reproductive success with its specific main ant visitor species.

To investigate if the number of floral buds and seeds per branch were influenced by the aggressiveness of the main ant attendant and by general ant visitation frequency, we built generalized linear mixed models (GLMMs) with Poisson distribution. In each model, we used as fixed factors: (1) the proportion of the total observations in which each focal plant was visited by ants; and (2) the aggressiveness category (low, intermediate or high) of the main ant visitor. Since all data was collected in three large areas within the study site, area identity was included as a random effect in all models. As the number of fruits that produced seeds in each branch influences the total number of seeds per branch, we used the number of fruits that ripened in each branch as weights in the models in which number of seeds were the response variable.

EFN-attending ants may influence not only plant investment in reproductive structures but also the proportion of such structures that remain viable through reproductive phase (e.g. [38]). Hence, we also analysed the proportion of floral buds that turned into flower, the proportion of flowers that turned into fruits and the proportion of fruits that ripened in plants attended in variable frequency by different ant species. For these analyses, we used GLMMs with binomial distribution, using the same set of fixed and random factors used in the analyses described above. We excluded individuals that did not produced flowers from the analysis of flowers that turned into fruits, and individuals without fruits from the analysis of proportion of ripened fruits.

For all analysis described above, we calculate all the probabilities using likelihood ratio tests [39]. For this, we first compared the full model containing both predictor variables and their interaction with a null model. When this comparison was significant, we evaluated the effect of each predictor variable by comparing reduced models (each reduced model lacking the interaction between variables or only one predictor variable) with the full model. We performed all analyses using lme4 package [40] in R software (R Core Development Team 2014). If our hypothesis is correct, we expect that an increase in ant visitation frequency when the main ant attendant is more aggressive will result in (1) the production of more floral buds; (2) in a higher proportion of floral buds turned into flowers, (3) of flowers turned into fruits, (4) of fruits that ripened; and/or (5) in the production of more seeds per branch.

## Results

### Ant aggressiveness

We observed 14 ant morphospecies visiting the baits (Table 1) and from those, only *Crematogaster* sp., *Pheidole* sp.2, *Acromyrmex* sp. and *Dinoponera quadriceps* were not observed attending EFNs. From the total 10 ant morphospecies observed on the focal plants, only *Solenopsis* sp., *Ectatomma bruneum*, *Camponotus blandus*, *Dorymyrmex piramicus*, and *Pheidole* sp.1 were observed interacting with a focal plant at least 60% of the time in which the focal plants were attended by ants. The more aggressive morphospecies were not the most frequent main visitor species: *Solenopsis* sp. (aggressiveness (A) = 1.35; N = 4 plants), *Ectatomma bruneum* (A = 0.64; N = 8 plants), *Camponotus blandus* (A = 0.35; N = 53 plants), *Dorymyrmex piramicus* (A = 0.23; N = 40 plants) and *Pheidole* sp. 1 (A = 0.12; N = 5 plants). There was no correlation between the frequency of observation of each ant morphospecies on focal plants and its aggressiveness score (Pearson correlation, r = 0.32, p = 0.26). On the other hand, aggressiveness was positively correlated with the mean number of individuals recruited to the baits per observation (r = 0.83, p < 0.001). We categorized *Solenopsis* sp. and *E*. *bruneum* as ants exhibiting “high aggressiveness” (N = 12 plants), C. blandus as “intermediate aggressiveness” (N = 53), and *D*. *piramicus* and *Pheidole* sp.1 as “low aggressiveness” (N = 45).

**Table 1:** Ant aggressiveness at study area. Ant species attracted by soil baits in three Caatinga areas at Federal University of Feira de Santana–Northeastern, Brazil.

Species	Ranking of aggressiveness	Number of observations at baits	Average number of individuals per bait	Aggressiveness degree	Aggressiveness category
Sub-Family Dolichoderinae					
Dorymyrmex piramicus *	4°	481	5.01	0.23	Low
*Tapinoma* sp.	8°	3	1	0	Low
Sub-Family Ectatomminae					
*Ectatomma* bruneum*	2°	119	1.39	0.65	High
*Ectatomma muticum*	9°	13	1.23	0	Low
*Gnamptogenys* sp.	9°	2	1	0	Low
Sub-Family Formicinae					
Camponotus blandus*	3°	277	6.53	0.36	Intermediate
*Camponotus substitutus*	7°	75	1.44	0.05	Low
*Camponotus* sp. 1	9°	105	2.09	0	Low
Sub-Family Myrmicinae					
*Crematogaster* sp.	8°	39	12.77	0.03	Low
*Solenopsis* sp. *	1°	156	36.09	1.35	High
*Pheidole* sp. 1*	5°	62	2.42	0.13	Low
*Pheidole* sp. 2	9°	37	0.11	0	Low
*Acromyrmex* sp.	9°	87	1.36	0	Low
Sub-Family Ponerinae					
*Dinoponera quadriceps*	5°	8	1	0.13	Low

“*Aggressiveness degree”* = score of aggressiveness of each ant species observed at the baits. This score was determined by the number of aggressive behaviours performed by individuals of each species by sharing the baits with other ant species. “*Aggressiveness category”* = category in which each ant species was classified based on their aggressiveness degree (low, intermediate and high).

* indicate the ant species that was the main attendant (>60% of the total visit by ants) of *Turnera subulata* (Turneraceae) plants in our study site.

### Influence of ant visitation on plant reproductive success

**Fig 1:**
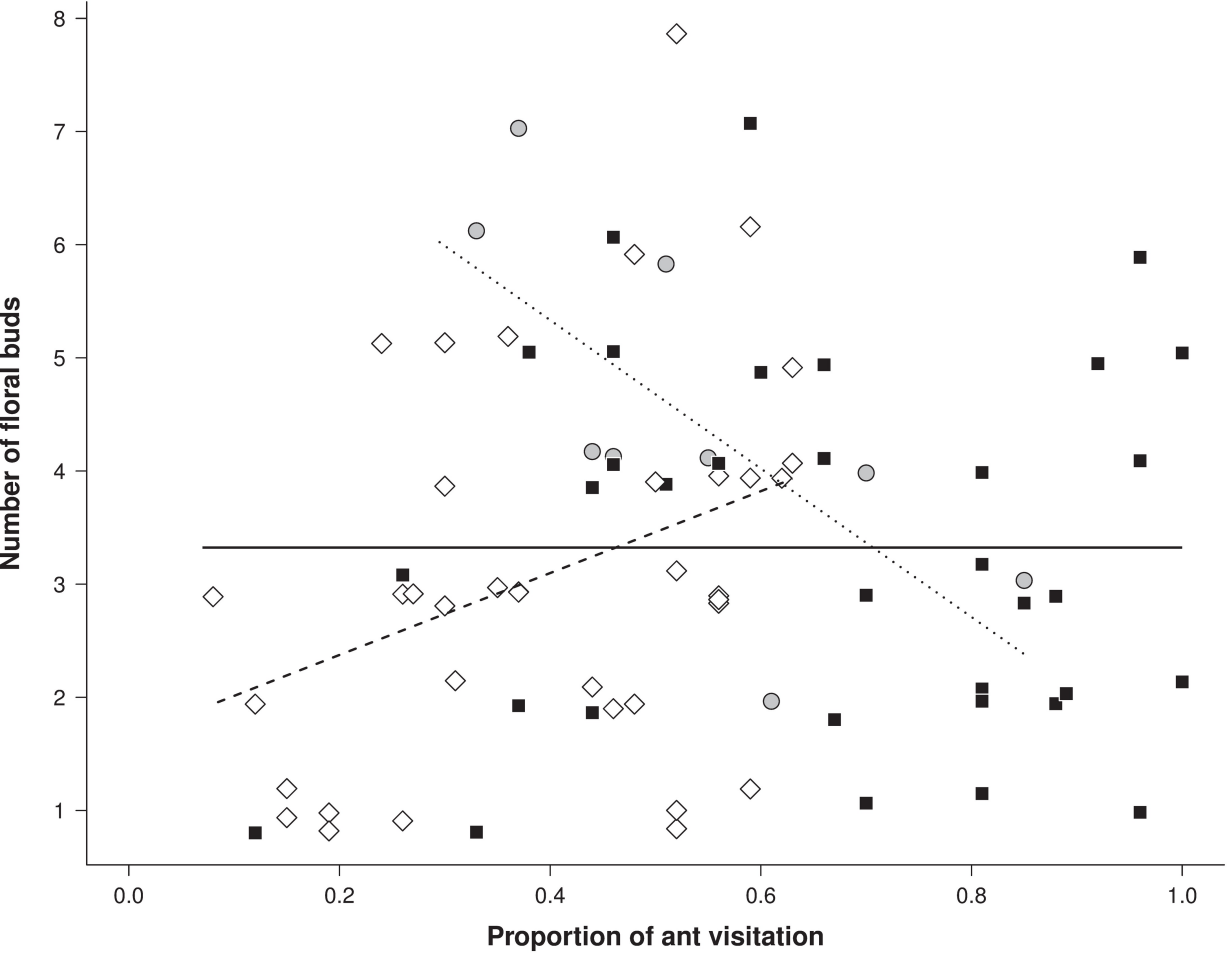
Number of floral buds as a function of the proportion of ant visitation and ant aggressiveness. Number of floral buds produced per branch of the EFN-bearing plant *Turnera subulata* (Turneraceae) visited in variable frequency by ants differing in its aggressiveness degree. The main ant species attending the EFN’s in each plant (> 60% of the total observed visitation) was classified into three groups according to its aggressiveness towards soil baits: low (squares and continuous line; *Pheidole* sp. and *Dorymyrmex piramicus*), intermediate (rhomb and dashed line; *Camponotus blandus*) and high aggressiveness (circle and dotted line; *Ectatomma bruneum*. and *Solenopsis* sp.). Low aggressiveness: Intercept = 3.32, Estimate = -0.00003. Intermediate aggressiveness: Intercept = 1.65, Estimate = 3.61. High aggressiveness: Intercept = 7.95, Estimate = -6.56.

Branches of the focal plants presented on average 2.44 ± 2.06 (mean ± SD) flower buds, 1.70 ± 1.73 flowers, 1.29 ± 1.41 fruits, 0.9 ± 1.20 ripe fruits and 19.15 ± 16.80 seeds. Additionally, 49% ± 0.42 floral buds turned into flowers, 47% ± 0.44 flowers turned into fruits and 43% ± 0.45 fruits ripened until seed dispersion. For the number of floral buds, the full model indicated that proportion of ant visitation, aggressiveness of main ant visitor and their interaction influenced the number of floral buds produced per branch of each focal plant (χ^2^ = 10.21, df = 5, *P* = 0.007). Removal of the interaction term from the models affected model explicability (χ^2^ = 5.91, df = 2, *P* = 0.04), suggesting that interaction between interaction frequency and aggressiveness of the main ant visitor influences the number of floral buds produced by plants. Increasing in the interaction frequency with ants when the main attendant was a less aggressive species (*Pheidole* sp.1 and *Dorymyrmex piramicus*) did not affected the number of floral buds (Z = 1.61, *P* = 0.10) (Fig 1). However, variation in floral buds number in response to increase in the frequency of EFN attendance when main ant visitor was intermediate or highly aggressive resulted in opposite patterns. Increasing in the frequency of plant attendance by ants was positively related to the number of floral buds per branch in plants in which the main ant visitor was an intermediate aggressive species (Z = 2.32, *P* = 0.02), and negative related to the number of floral buds when the main visitor was a highly aggressive ant species (Z = 2.43, *P* = 0.01) (Fig 1).

For the proportion of buds that turned into flowers, the explicability of the full model did not differ from the null model (χ^2^ = 8.76, df = 5, *P* = 0.11). Likewise, the explicability of full model for variation in the proportion of flowers turned into fruits and fruits that ripened were similar to the explicability of the null models (χ^2^ = 1.68, df = 5, *P* = 0.89; fruits ripened: χ^2^ = 3.75, df = 5, *P* = 0.58).

**Fig 2:**
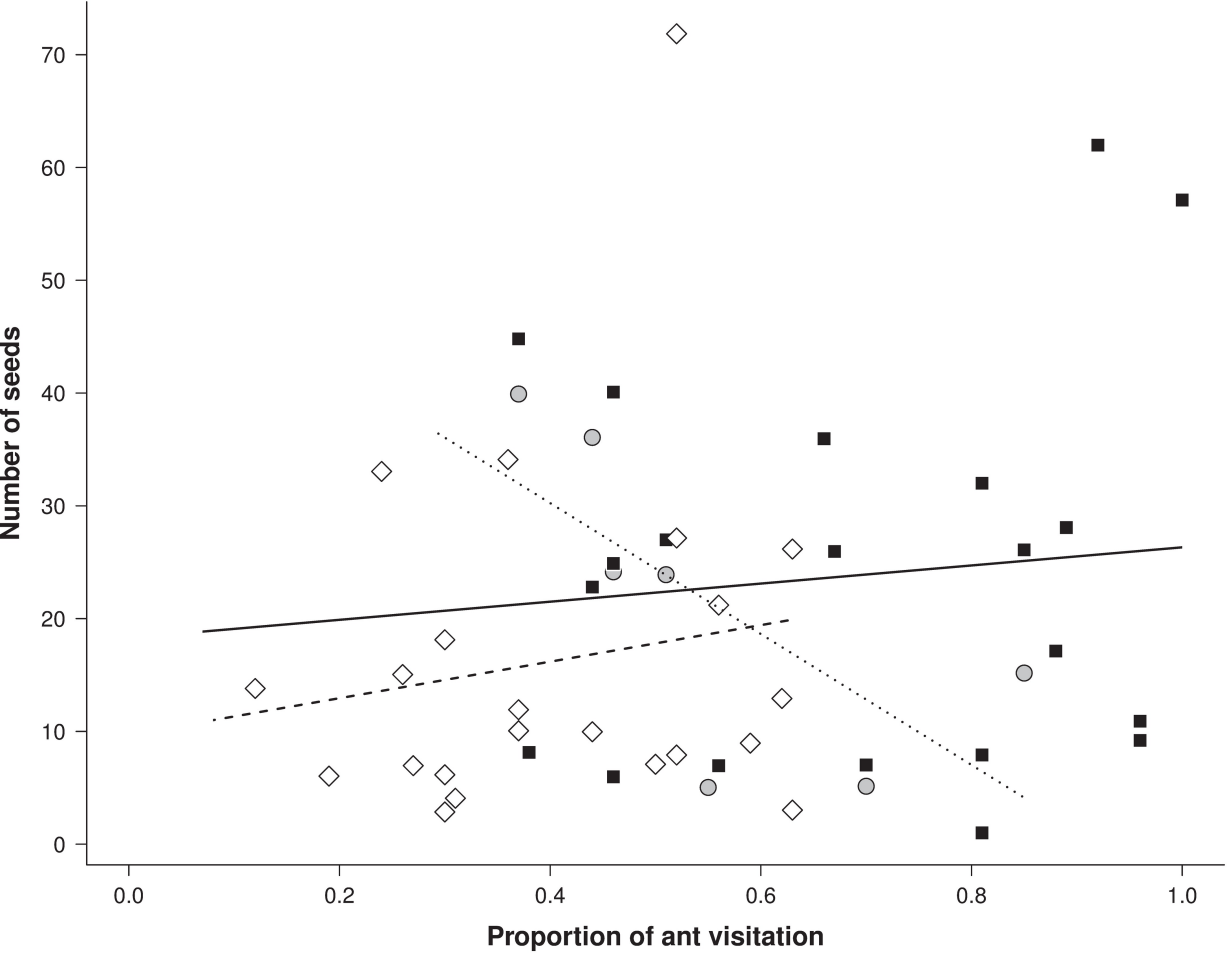
Number of seeds as a function of the proportion of ant visitation and ant aggressiveness. Number of seeds produced per branch of the EFN-bearing plant *Turnera subulata* (Turneraceae) visited in variable frequency by ants differing in its aggressiveness degree. The main ant species attending the EFN’s in each plant (> 60% of the total observed visitation) was classified into three groups according to its aggressiveness towards soil baits: low (squares and continuous line; *Pheidole* sp. and *Dorymyrmex piramicus*), intermediate (rhomb and dashed line; *Camponotus blandus*) and high aggressiveness (circle and dotted line; *Ectatomma bruneum* and *Solenopsis* sp.). Low aggressiveness: Intercept = 18.29, Estimate = 8.03. Intermediate aggressiveness: Intercept = 9.70, Estimate = 16.19. High aggressiveness: Intercept = 53.46, Estimate = -58.08).

The full model indicated that proportion of ant visitation, aggressiveness of main ant visitor and their interaction were associated to the number of seeds produced per branch in each focal plant (χ^2^ = 248.35, df = 5, *P* <0.001). Removal of interaction term from the models affected model explicability (χ^2^ = 150.92, df = 2, *P* < 0.001), suggesting that model including the interaction between interaction frequency and ant aggressiveness explained the variation in the number of seeds produced by plants. However, such pattern could be biased by three outliers formed by the plant with the highest number of seeds per branch. Removal of such potential outliers led to no qualitative changes in the result (full x null model: χ^2^ = 63.85, df = 5, *P* <0.001; removal of interaction term: χ^2^ = 52.93, df = 5, *P* <0.001). Overall, an increase in the visitation frequency when highly aggressive ants were the main visitor (*Ectatomma bruneum* and *Solenopsis* sp.) was associated with a decrease in the number of seeds produced per branch (Z = 8.28, *P* = 0.03) (Fig 2). On the other hand, an increase in the frequency of interactions when main ant visitors presented low (*Pheidole* sp.1 and *Dorymyrmex piramicus*) and intermediate (*C*. *blandus*) aggressiveness was associated with a slightly increase in the number of seeds produced (intermediate: Z = 8.14, *P* < 0.001; low: Z = 9.17, *P* < 0.001) (Fig 2).

## Discussion

We observed that neither the degree of aggressiveness of the main ant visitor nor the frequency of plant interaction with ants influenced the maintenance of the flowers and fruits through the reproductive cycle. Nevertheless, the interaction between ant visitation frequency and ant aggressiveness influenced the total number of floral buds and seeds produced per branch in the focal plants. This suggests that attending ants play a minor role in the maintenance of reproductive structures of *T*. *subulata* over time, but that the initial investment of *T*. *subulata* in reproductive structures and in the production of diaspores can be affected by the longevity of the association with ants that differ in their potential aggressiveness. However, contrary to our expectation, increasing in the frequency of interaction with highly aggressive ant species lead to a reduction in the number of flower buds and seeds produced by focal plants. Additionally, plants interacting more frequently with intermediate or low aggressive ants tended to benefit more from the interaction, producing more floral buds and more seeds per branch.

Although our measure of ant aggressiveness is based on ant behavior exploring soil baits, other study performed by our study group investigating a different question in the same study area indicates that ant aggressiveness on soil baits is related to its efficiency in removing potential herbivores from *T*. *subulata* (Passos & Leal, *in prep*). In this study, our highly aggressive ant species (*Solenopsis* sp.1 and *Ectatomma bruneum*) removed 80% of simulated herbivores (larvae of *Ulomoides dermestoides*; Coleoptera, Tenebrionidae) positioned on *T*. *subulata* plants. Meanwhile, our intermediate (*C*. *blandus*) and low (*Dorymyrmex piramicus* and *Pheidole* sp.1) aggressive ant species removed 73% and 65% of the mimics, respectively. Hence, even though more aggressive ant species can be more efficient plant bodyguards [20, 30, 31], it is possible that long-term interaction with the most aggressive ants available in the environment may impose some costs that can be detrimental to plant fitness.

In the case of aggressive ants, increasing in the interaction cost with increasing in frequency of interaction may be caused by two non-mutually-exclusive mechanisms acting in distinct periods of plant lifetime. First, ants of different species can impose different direct costs when consuming the extrafloral nectar. A recent study, [20] evaluating ant response to manipulation in the quality of artificial extrafloral nectar showed that more aggressive ant species dominate plants in periods of high quality nectar secretion. Additionally, during our measures of ant aggressiveness, we observed that ant aggressiveness was positively correlated to the number of recruited workers to the baits. Aggressiveness among ant species is a behavioural trait correlated to other traits as group foraging, recruitment of large number of individuals to food sources and larger ant nests [26, 28]. Because of it, EFN-plants monopolized by more aggressive ants have higher probability to shelter a higher number of individuals consuming extrafloral nectar over time in comparison with plants frequently interacting with less aggressive ant species. Altogether, although extrafloral nectar secretion is considered physiologically cheap [41,42]), it is possible that the maintenance of a long-term interaction with more aggressive ants require higher plant investment in nectar secretion, relatively increasing the interaction costs to such plants. If so, reduction in plant investment in reproductive structures and/or seed production may offset such increased costs of interaction with ants. Such costs imposed by more aggressive ant species can be especially high in situations in which herbivory pressure is low. In such scenario, the loss of photosynthetic leaf area due to herbivore consumption is low, making the costs imposed by more aggressive bodyguards relatively higher in comparison to scenarios in which the cost of interacting with bodyguards that are more aggressive can be compensated by increasing in photosynthetic rate due to reduction in leaf area lost to herbivores. Therefore, when herbivore pressure is low, plants interacting more frequently with less aggressive (and cheaper ants) would perform relatively better than the ones monopolized by more aggressive (and costly) ants. During our experiments, we have found no herbivores naturally foraging on our focal plants. It suggests that our plants were submitted to low herbivore pressure in our study site, reinforcing the idea that more aggressive ants can be physiologically costlier to EFN-plants than less aggressive ones in such scenarios.

A second mechanism that may be acting more directly on the number of seeds produced in each focal plant is the effect of different ant visitors on the pollination of EFN-bearing plants. The indirect negative effect of ant bodyguards on pollination of plants exhibiting biotic defenses is an already known ecological cost associated to this kind of ant herbivore defence [6, 30, 43, 44], including for EFN-bearing *Turnera* species [45]. However, the aggressiveness of ant species attending the EFN has seldom being considered as a potential factor regulating such costs (but see [30]). If on the one hand more aggressive ant species are more efficient expelling plant herbivores, on the other they may also be more aggressive against potential plant pollinators, especially in the case of EFN-bearing plants like *T*. *subulata* in which EFNs are located close to the flowers. Therefore, the influence of more aggressive ant species on the identity and behaviour of pollinators may directly influence pollination success, potentially reducing the number of seeds formed at the end of the reproductive cycle. Otherwise, less aggressive ants may have lesser influence over the identity and behaviour of pollinators. Unfortunately, our data did not allow us to investigate the direct (e.g. cost related to EFN consumption) and indirect costs (e.g. negative effect on pollination success) imposed by different ant groups to our focal plants. However, we suggest that such two mechanisms can be fruitful venues for future investigations regarding the effect of different partner species on the outcome of this facultative ant-plant mutualism.

Finally, we are aware that our measurements of plant reproductive success can result mostly from previous interactions between the focal plants and the local ant assemblage. As ants and plants are both sessile organisms, their probability of interaction over time is mainly determined by their spatial distribution [38]. As we have no reason to believe that plant and ant species distribution on our study site have changed in the recent years, we argue that the visitation frequency we measured is a good estimate of the previous pattern of ant visitation on the focal plants in our study area. Additionally, if how ants use plants changes with plant quality over time, then our results may be biased. EFN activation (and consequently, its quality to ants) is directly related to plant phenology and each individual plant has its phenology triggered differently passing years [46,47]. If aggressive ants tend to be the first ones to monopolize plants that have early EFN activation, plant reproductive success could be a simple consequence of patterns of plant monopolization by ant species with different aggressiveness over time. However, if this would be happening in our study, we should have found an opposite pattern, in which plants interacting in higher frequency with more aggressive ants had higher reproductive success than the ones monopolized by less aggressive ants. According to our results, even if aggressive ants are monopolizing more vigorous plants with early EFN activation in our study site, such monopolization is leading to a decreasing in the reproductive success of these early phenology plants.

## Conclusion

Our results indicate that although these aggressive ant species can provide better ‘momentary’ benefit to plant pattern, engaging in a long-term interaction with them can incurs in increasing cost to the plants, reducing the plant reproductive success. We propose that such costs may arise mainly through increasing in nectar consumption by aggressive ants that are commonly more abundant on the surface of monopolized plants and/or by reduction in pollination efficiency due to repellence of pollinators by ants that are more aggressive. Putting these conclusions in a broader perspective, our results highlight that the quality of a mutualistic partners must be understood as a function of its per-interaction benefit [efficiency] and their cumulative costs to their partner over time in different ecological scenarios. For that, it is necessary to incorporate temporal aspects in the study of mutualistic interactions in general, evaluating the interplay between costs and benefits that each species provide to its partner through time.
